# Dual-optimization hypothesis: integrating radiation dosimetry and mechanical reinforcement in radioactive seed implantation for lumbar metastases

**DOI:** 10.3389/fonc.2026.1769562

**Published:** 2026-03-10

**Authors:** Zhi-qian Sun, Lu-lu Du, Shuai Li, Guangyan Gao, Qi-yu Sun, Bao-quan Zhu, Yue-feng Cai, Chen Zhong, Min Li

**Affiliations:** 1 Department of Nuclear Medicine, The 960th Hospital of the PLA Joint Logistic Support Force, Jinan, Shandong, China; 2 School of Medical Imaging, Shandong Second Medical University, Weifang, Shandong, China; 3 The Postgraduate Training Base of Jinzhou Medical University (The 960th Hospital of Chinese People's Liberation Army (PLA)) , Jinan, China; 4 University of Illinois Urbana-Champaign, Urbana, IL, United States; 5 Department of Medical Oncology, The 960th Hospital of the Chinese People's Liberation Army (PLA) Joint Logistic Support Force, Jinan, Shandong, China

**Keywords:** dual-optimization hypothesis, lumbar metastases, mechanical reinforcement, pain relief, radiation dosimetry, radioactive seed implantation

## Abstract

**Objective:**

To explain the rapid pain relief observed hours after radioactive seed implantation for lumbar metastases (which precedes radiobiological effects) and propose a novel therapeutic framework that integrates two core functions of titanium-encased radioactive seeds: delivering therapeutic radiation and providing immediate mechanical reinforcement to compromised vertebrae.

**Methods:**

A nonlinear finite element analysis (FEA) was conducted on an L4-L5 vertebral metastasis model to quantify the biomechanical effects of seed implantation. The analysis focused on changes in cortical bone stress peaks and load redistribution patterns in fracture-prone zones, while correlating seed activity levels with implantation density, spatial distribution, dosimetric coverage, and biomechanical reinforcement effects.

**Conclusion:**

Finite element simulations in a patient-specific L4–L5 model indicate titanium-encased seed implantation reduces cortical stress peaks (16.2% in this model) and redistributes loads from fracture-prone regions. These mechanical changes align with immediate stabilization, potentially aiding early pain relief—though causality cannot be established, as pain is multifactorial and our model only addresses mechanical aspects. We thus propose a dual-optimization framework integrating TPS-based dosimetry with biomechanical objectives to inform both short-term stabilization potential and long-term radiobiological control. Within the scope of the present L4–L5 case, this integrated TPS–biomechanics framework provides a hypothesis-driven approach to optimize implantation planning, while extension of quantitative findings to other spinal levels requires dedicated modeling and validation.

## 1 Introduction

lumbar metastases present a complex clinical challenge, with pain as the predominant symptom severely affecting patient quality of life. Radioactive seed implantation has proven effective for treating these lesions, with conventional understanding attributing outcomes exclusively to radiation-induced tumor control (1). The standard paradigm suggests that pain relief should follow the timeline of radiation effects, typically requiring weeks to manifest (2).

However, a striking clinical observation challenges this understanding: many patients report substantial pain reduction within hours or a single day following seed implantation—a phenomenon that cannot be explained by radiation effects alone. This temporal discrepancy represents a significant knowledge gap in our understanding of the therapeutic mechanisms involved.

The current approach to radioactive seed therapy focuses exclusively on optimizing radiation dose distribution through treatment planning systems (TPS). Seeds of varying activities are selected and positioned to achieve target dosimetric coverage while sparing critical structures. This approach overlooks the potential biomechanical impact of these metallic implants within the compromised vertebral structure.

Our previous research has demonstrated that even limited volumes of implanted material can significantly restore mechanical integrity to metastatic vertebrae. This finding raises the compelling possibility that radioactive seeds, beyond their radiation effects, function as a distributed internal stabilization system within the vertebral body.

## 2 Hypothesis

We hypothesize that radioactive seeds implanted for vertebral metastasis treatment serve a dual function: delivering therapeutic radiation while simultaneously providing immediate mechanical reinforcement to compromised vertebral structures. The variation in seed activities, which determines the number and spatial distribution of implanted seeds, directly influences both radiation dose delivery and the architecture of mechanical reinforcement within the vertebral body. The titanium-encased seeds create a three-dimensional support network that immediately stabilizes the vertebral structure, explaining the early pain relief phenomenon observed clinically. Different patterns of seed distribution, necessitated by varying seed activities in dosimetric planning, create distinct biomechanical reinforcement architectures with differentiable effects on vertebral stability. We believe that optimal therapeutic outcomes require simultaneous consideration of both dosimetric coverage and mechanical reinforcement patterns in treatment planning.

## 3 Evidence supporting the hypothesis

### 3.1 Clinical evidence

The clinical observation of pain relief within hours post-implantation contradicts the established timeline of radiation effects. This temporal disconnect strongly suggests an alternative mechanism for immediate pain reduction, consistent with mechanical stabilization effects. Studies in vertebroplasty have demonstrated that the spatial distribution of bone cement significantly impacts the mechanical restoration achieved (3). Similarly, the three-dimensional arrangement of radioactive seeds—determined by dosimetric requirements and seed activities—likely creates varying patterns of mechanical support within the vertebral body.

The selection of radioactive seeds with different activities directly determines the number of implants and their spatial distribution required to achieve target dosimetric coverage: higher-activity seeds allow for fewer implants with larger spacing, while lower-activity seeds require more implants with dense arrangement. These distinct distribution patterns not only affect dosimetric coverage but also form different mechanical reinforcement architectures, which in turn result in differences in the timing and degree of symptom relief observed in patients (see **Table 1** for details).To verify the correlation between seed activity, distribution pattern and pain relief, this study retrospectively analyzed data from 96 patients with lumbar metastases. Among the 96 patients, 41 were assigned to the high-activity group (0.8 mCi) and 55 to the low-activity group (0.3-0.6 mCi).There were no statistically significant differences in age, gender distribution, type of primary tumor, preoperative fracture status, and preoperative pain severity (P > 0.05). The preoperative VAS score of patients in the high-activity group (0.8 mCi) was 7.84 ± 0.802. It dropped to 6.03 ± 0.97 at 9 hours post-operation and to 5.85 ± 0.85 at 16 hours post-operation (P < 0.001). The preoperative VAS score of patients in the low-activity group (0.3 - 0.6 mCi) was 7.81 ± 0.86. It dropped to 5.75 ± 0.76 at 9 hours post-operation and to 5.66 ± 0.81 at 16 hours post-operation (P < 0.001). The comparison between the groups showed that the VAS score at 9 hours post-operation in the low-activity group was significantly lower than that in the high-activity group, and the difference was statistically significant (P < 0.05); while there was no significant statistical difference in the VAS score at 16 hours post-operation between the two groups (P > 0.05, **Table 2**). This may indicate that the low-activity group had a more significant immediate pain relief (larger VAS score reduction) due to a greater number of implanted particles and a more dense distribution, which is consistent with the biomechanical rationale that a denser distribution may provide more uniform mechanical support. This finding is consistent with the possibility that mechanical reinforcement contributes to immediate pain relief.

**Table 1:** Correlation between seed activity, implantation parameters, and symptom relief outcomes in radioactive seed implantation for lumbar metastases.

Prescription dose range (Gy)	Seed activity (mCi)	Number of implants	Distribution pattern (Unilateral/Bilateral)	Symptom relief time	Relief degree
140-160	0.8 (High)	8-12	Predominantly unilateral, relatively dispersed	8-24 hours	Moderate
140-160	0.3-0.6 (Low)	15-25	Bilateral or unilateral dense distribution	6-12 hours	Significant

**Table 1** provides a descriptive (illustrative) summary based on retrospective clinical observations and standard dosimetric planning logic; it is not intended to imply quantitative causality.

**Table 2:** Comparison of VAS scores between the two groups of patients at different time points before and after the operation.

Group	Preoperative VAS	Postoperative 9 hours VAS	Postoperative 16 hours VAS	P value
High activity group	7.84 ± 0.80	6.03 ± 0.97	5.85 ± 0.85	<0.001
Low activity group	7.81 ± 0.86	5.75 ± 0.76	5.66 ± 0.81	<0.001
P value	>0.05	<0.05	>0.05	

**Figure 1:**
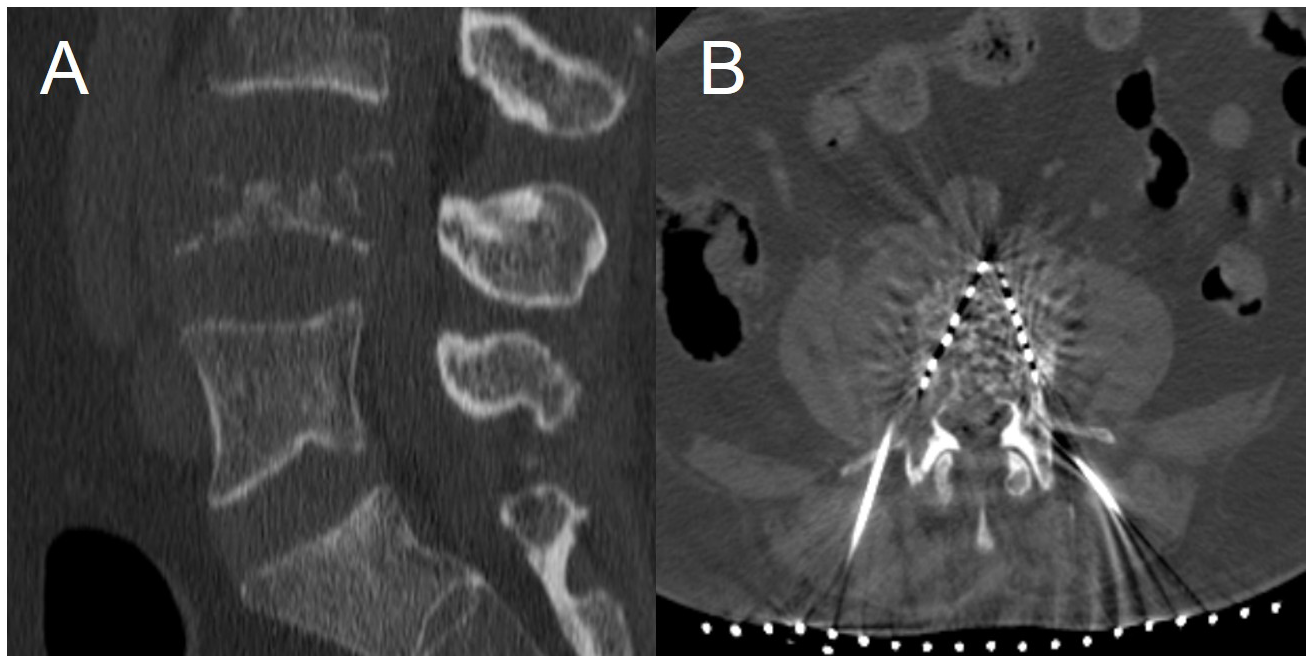
A 65-year-old male patient with lung cancer and L4 vertebrae bone metastasis. **(A)** The CT sagittal view shows that there is osteolytic bone destruction in the L4 vertebra. **(B)** A 125-I particle implantation procedure for the L4 vertebra was performed under CT guidance. The axial view showed that the 125-I particles were evenly distributed, and the patient experienced a significant immediate pain relief.

Titanium alloy possesses high compressive strength and elastic modulus (4). The collective reinforcement effect of multiple seeds strategically distributed throughout the metastatic region provides immediate structural support, similar to other vertebral augmentation techniques. (**Figure 1**).

### 3.2 Biomechanical evidence from finite element simulation

**Figure 2:**
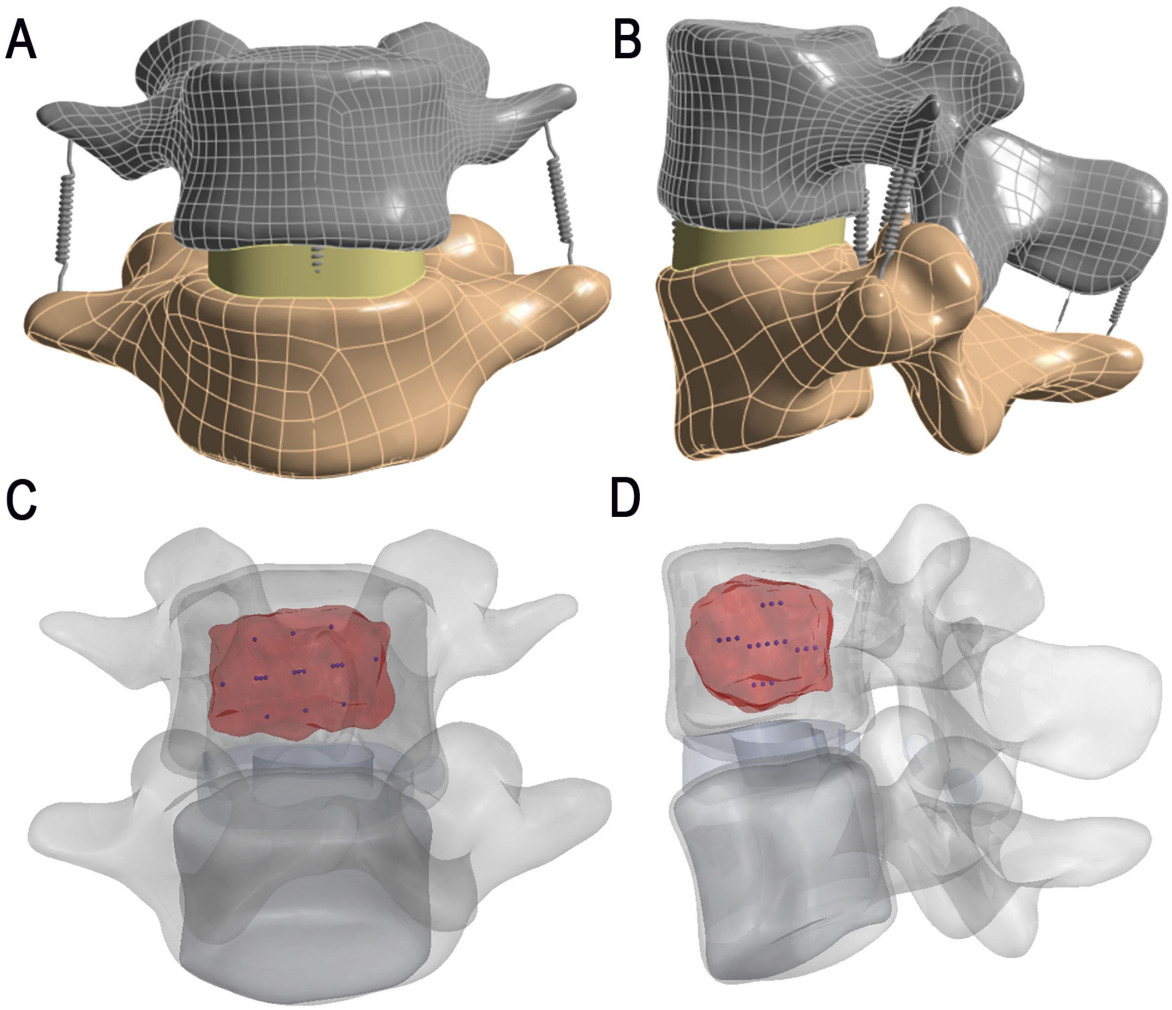
Finite element model of the L4–L5 spinal segment with 125-I seed implantation. **(A, B)** Anterior and lateral views of the L4–L5 segment showing the segmented cortical bone (gray), trabecular bone (yellow), intervertebral disc (green), and posterior elements. **(C, D)** Superior and oblique views illustrating the distribution of titanium-encased 125-I radioactive seeds (blue) implanted within the metastatic lesion (highlighted in red) in the L4 vertebral body.

Finite element analysis was conducted on a metastatically involved L4 vertebra (**Figure 2**) to quantify mechanical reinforcement. The cortical bone, articular cartilage, and bony endplates were assigned thicknesses of 1.5 mm, 0.3 mm, and 0.5 mm, respectively (5). Intervertebral discs were differentiated into nucleus pulposus and annulus fibrosus components, with the nucleus pulposus occupying 30%–40% of the disc volume. Spring elements were implemented to represent seven key spinal ligaments: the anterior longitudinal ligament (ALL), posterior longitudinal ligament (PLL), ligamentum flavum (LF), intertransverse ligaments (ITL, bilateral), interspinous ligament (ISL), and supraspinous ligament (SSL, 6).The facet joints between the vertebrae were considered as face-to-face contact with Coulomb friction and a friction coefficient of 0.1 (6). All other interface contact types were classified as “bonded”. All degrees of freedom of nodes on the inferior surface of the L5 vertebra were constrained. A uniform downward load of 500 N was applied to the superior surface of the L4 vertebra to simulate upper body pressure under physiological conditions (7).Mesh sensitivity analysis was performed using the Maximum von Mises stress in the L4 cortical shell and the L4 superior endplate as indicators, with the characteristic element size refined from 1.0 mm to 0.6 mm. When the mesh size was ≤ 0.8 mm, discrepancies in these key responses relative to the 0.6 mm mesh were below 5%. A mesh size of 0.6 mm was therefore adopted for subsequent analyses. The model range of motion (ROM) in flexion, extension, lateral bending, and axial rotation fell within the ranges reported in previous experimental and finite element studies (8–10) (**Table 3**).

**Table 3:** Comparison of L4-L5 range of motion (ROM) in the intact finite element model of this study with previously reported data.

Loading condition	L4-L5			
	Present Study	Li	Renner	Panjabi
Flexion/Extension	8.3	1.9	13.75	11.5
Lateral Bending	7.6	4.7	10.35	8.3
Axial Rotation	3.2	2.9	3.9	1.8

For consistency with prior spine FEA studies and to reduce parameter uncertainty given limited patient-specific material data for metastatic lesions and disc degeneration, all tissues (bone, tumor, and disc components) were modeled as linear elastic and isotropic. This simplification was adopted to support comparative assessment between pre- and post-implantation models under identical loading, rather than to provide absolute failure predictions. Case-specificity. This FEA was performed on a single patient-specific L4–L5 segment; thus, stress magnitudes and percentage changes are case- and level-specific and should not be generalized beyond the modeled segment.

Comparative analysis revealed:

Baseline (pathological) model: Stress concentrations at the vertebral endplates reached 20.92 MPa (von Mises), exceeding that of the healthy group (15 MPa), indicating stress concentration due to the presence of metastatic lesions.

**Figure 3:**
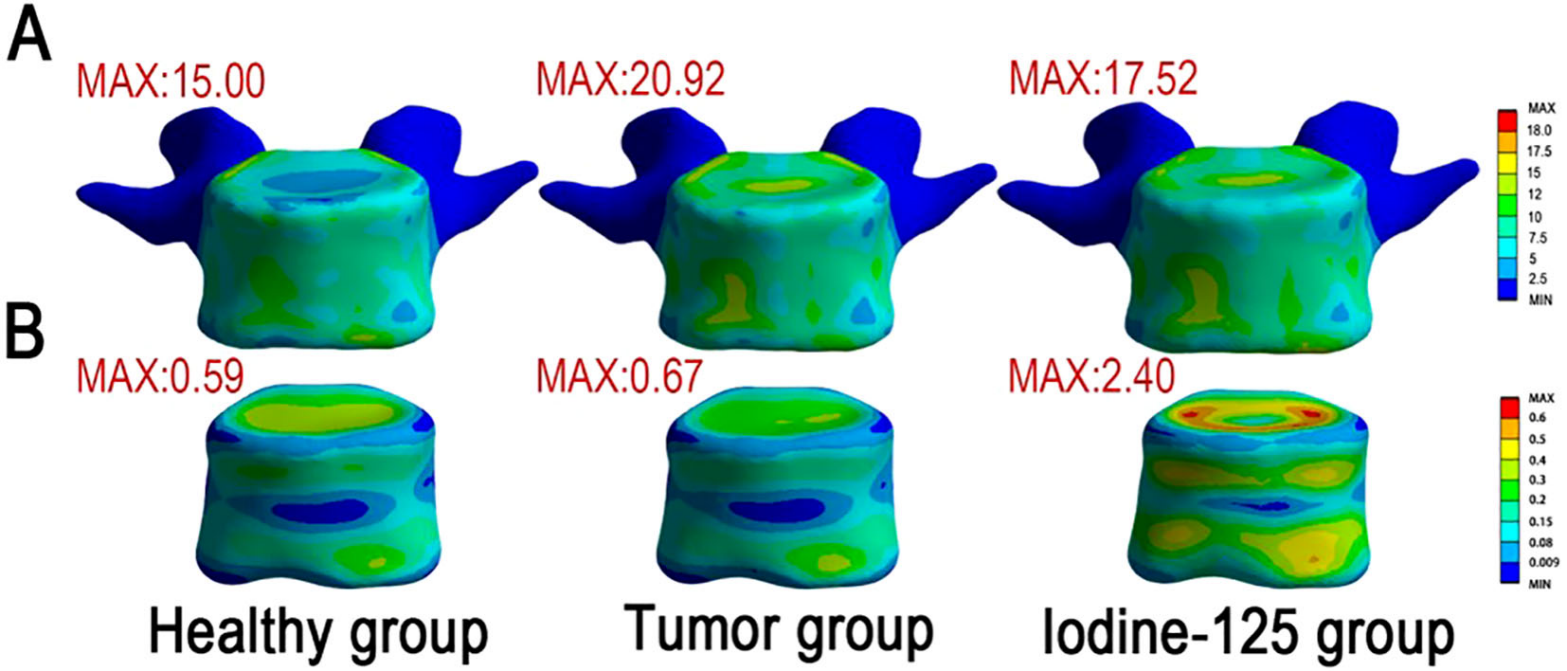
von Mises stress distribution in vertebral models across healthy, tumor, and 125-I implantation groups. **(A)** The stress distribution in the cortical bone revealed that the tumor group exhibited a higher peak von Mises stress (20.92 MPa) compared to the healthy group (15.00 MPa), indicating stress concentration due to the presence of metastatic lesions. Following iodine-125 seed implantation, the peak stress decreased to 17.52 MPa, suggesting a partial mitigation of stress concentration and a more favorable biomechanical environment. **(B)** In the cancellous bone (metastatic region), the tumor group showed slightly elevated local stress relative to the healthy group (0.67 MPa vs. 0.59 MPa). Notably, the 125-I implantation group demonstrated a substantial increase in peak stress to 2.40 MPa, implying a redistribution of local mechanical loads and a marked alteration of the stress environment as a result of brachytherapy.

Post-implantation model: Titanium seeds (material properties in **Table 4**) reduced peak cortical stresses by 16.2% to 17.52 MPa (**Figure 3A**). Cancellous bone stress increased by 260% (0.67→2.40 MPa; **Figure 3B**), indicating load transfer from cortical regions to the seed-reinforced trabecular compartment. This increase may be sensitive to linear-isotropic material assumptions and should be interpreted as a relative inter-model change, not an absolute failure metric.

**Table 4:** Material properties for finite element analysis of the lumbar L4-L5 metastasis model.

Material	Young’s Modulus (MPa)	Poisson’s Ratio	Cross-Sectional Area (mm²)
Cortical Bone	12,000	0.30	-
Trabecular Bone	132	0.20	-
Annulus Fibrosus	4.2	0.45	-
Nucleus Pulposus	1	0.499	-
Metastatic Tumor	1	0.45	-
Ti6Al4V	144,000	0.32	
Articular Cartilage	23.8	0.40	-
Anterior Longitudinal Ligament	20	0.30	65
Posterior Longitudinal Ligament	20	0.30	20
Ligamentum Flavum	19.5	0.30	40
Intertransverse Ligament	59	0.30	1.8
Supraspinous Ligament	15	0.30	30
Interspinous Ligament	12	0.30	40

These findings suggest two biomechanical trends (1): partial stress shielding of weakened cortical bone through local stiffening, and (2) reduced stress risers via altered load-path redistribution. Collectively, these changes may improve the mechanical microenvironment (e.g., lowering stress concentration and micromotion), providing biomechanical plausibility for an immediate stabilization component that could contribute to early pain relief; however, this simulation does not model microcrack dynamics or nociceptor activity and therefore does not establish a causal analgesic mechanism.

### 3.3 Hypothesis boundaries and limitations

To avoid overinterpretation, we emphasize that the dual-optimization hypothesis is supported by biomechanical plausibility rather than causal proof. Pain is multifactorial, and our model addresses only the mechanical component, not inflammatory or neurochemical drivers. The FEA results (including a 16.2% cortical stress-peak reduction in this model) provide supportive numerical evidence but do not establish a stress–pain causal link, as no validated pain–stress threshold or biomechanical surrogate of analgesia was applied. The simulations also represent a single anatomy/loading condition with simplified assumptions and do not capture patient heterogeneity or time-dependent remodeling. Finally, early pain relief may reflect synergistic non-mechanical factors (e.g., perioperative medications) and retrospective clinical data may be confounded; prospective validation is therefore needed. Material modeling simplification. Trabecular bone anisotropy, disc viscoelasticity, and metastatic heterogeneity were not represented; these factors could change the absolute stress magnitudes and local redistribution patterns. Nevertheless, because both baseline and post-implantation models used the same constitutive assumptions and loading, the reported stress changes are primarily intended for within-model comparison to reflect mechanical trends. More advanced formulations (e.g., orthotropic/anisotropic bone, poroelastic or viscoelastic disc models, and heterogeneous tumor properties) may alter the quantitative values (e.g., the reported trabecular stress amplification) but are unlikely to overturn the qualitative trend of load-path redistribution induced by stiff titanium inclusions under the same boundary conditions. Unilateral vs bilateral and dense vs sparse distributions were not parameterized/quantified in FEA; this should be tested in future parametric simulations.

## 4 Potential mechanisms

The proposed dual-optimization mechanism operates through several interconnected pathways. The three-dimensional network of titanium-encased seeds reduces pathological micromotion at the bone-tumor interface, immediately decreasing nociceptive stimulation. Different seed activities require different implantation patterns to achieve target dosimetry, creating varying mechanical support architectures. Higher activity seeds necessitate fewer implants with greater spacing, creating a more dispersed structural support pattern. Lower activity seeds require more numerous, closely-spaced implants, creating a more uniform reinforcement network.

The implanted seeds may redirect compressive and tensile forces away from pain-sensitive structures, consistent with stabilization mechanisms discussed in vertebroplasty (11). Early pain relief may therefore reflect an immediate mechanical stabilization component, whereas radiation gradually contributes to tumor control and sustained analgesia over subsequent weeks. In the present study, variation in seed activity can be viewed as a means to adjust both dosimetric coverage and the mechanical reinforcement architecture within the patient-specific L4–L5 model under the specified loading condition, without implying direct quantitative extrapolation to other spinal regions.

## 5 Clinical implications

The proposed dual-optimization framework may inform hypothesis-driven planning concepts for L4–L5 lumbar metastasis cases. Treatment planning could integrate biomechanical objectives with dosimetric goals—particularly for vertebrae with >30% cortical involvement where fracture risk escalates exponentially (12). Application to cervical or thoracic vertebrae requires region-specific FEA validation due to anatomical and loading differences. For mechanical pain predominance, seed distributions should maximize endplate stress reduction (suggested by FEA pre-planning). Conversely, neuropathic presentations may prioritize dosimetric coverage of neural structures. Critically, seed activity selection becomes a multivariable decision: high-activity seeds minimize cortical penetration but provide limited reinforcement, whereas low-activity configurations enhance stabilization at the cost of increased insertion trajectories. Emerging solutions include hybrid activity protocols and patient-specific FEA-guided planning algorithms.

## 6 Future directions

If confirmed, our hypothesis points toward a transformative approach to treatment planning for lumbar metastases. Development of integrated software that merges traditional TPS capabilities with finite element analysis could simultaneously model radiation dose distribution and mechanical reinforcement effects. Creation of algorithms that identify implantation patterns achieving both adequate dosimetric coverage and optimal mechanical support distribution would revolutionize treatment planning.

Incorporation of patient-specific vertebral structure, tumor involvement patterns, and biomechanical requirements into the planning process would allow truly personalized medicine approaches. Implementation of machine learning approaches could predict optimal seed activities and distributions based on both dosimetric and mechanical objectives. Development of specialized implants that optimize both radiation delivery and mechanical support functions, potentially through modified shapes or materials, represents another promising avenue for research.

## 7 Testing the hypothesis

Our hypothesis can be tested through several complementary approaches. Biomechanical studies should include evaluation of mechanical reinforcement provided by different seed distribution patterns resulting from varying seed activities. Computer simulation comparing the mechanical stability achieved with different implantation patterns dictated by varying seed activities would provide theoretical validation. Mechanical testing of vertebral specimens with metastatic disease before and after implantation with different seed activities and distribution patterns would offer empirical evidence.

Clinical investigations should include prospective studies correlating immediate pain relief with seed activity levels, quantities, and distribution patterns. Randomized trials comparing pain outcomes between standard dosimetric-optimized implantation and dual-optimized (dosimetric and mechanical) implantation would provide definitive evidence of clinical benefit. Detailed analysis of pain relief timing in relation to different seed activity levels and implantation patterns could further elucidate the mechanisms involved.

Development and validation of software integrating TPS and biomechanical modeling represents a critical step toward clinical implementation. Comparison of predicted mechanical and dosimetric outcomes with actual clinical outcomes would validate this approach. Evaluation of various optimization approaches to identify those that most effectively balance dosimetric and mechanical objectives could further refine this emerging treatment paradigm.

## 8 Conclusion

The dual-optimization hypothesis offers a novel explanation for the puzzling clinical observation of immediate pain relief following radioactive seed implantation for lumbar metastases. By recognizing that seed activity levels directly influence both radiation delivery and mechanical reinforcement patterns, we provide a comprehensive framework for understanding the full therapeutic mechanism of this treatment approach.

This integrated perspective challenges the conventional radiation-only paradigm and suggests a path toward truly optimized treatment planning that considers both the immediate mechanical and long-term radiobiological effects of seed implantation. The future of L4-5 vertebral metastasis treatment may lie in sophisticated planning systems that merge dosimetric calculations with patient-specific biomechanical modeling to achieve optimal outcomes in both pain relief and tumor control. Extension to cervical or thoracic regions will require region-specific validation accounting for anatomical, loading, and metastatic pattern differences.

If confirmed, this dual-optimization approach would represent a paradigm shift in radioactive seed therapy for L4-5 lumbar metastases, emphasizing the importance of considering the implanted seeds not merely as radiation delivery vehicles but as a sophisticated system providing both structural support and therapeutic radiation. The integration of biomechanical principles with traditional radiation oncology concepts opens new avenues for interdisciplinary research and treatment innovation for patients suffering from this challenging condition.

## Data Availability

The raw data supporting the conclusions of this article will be made available by the authors, without undue reservation.
